# The Book World of Medicine and Science

**Published:** 1901-09-14

**Authors:** 


### The Book World of Medicine and Science.

Report on the Bacteriological Diagnosis and the Antitoxic Serum Treatment of Cases Admitted to the Hospitals of the Metropolitan Asylums Board during the Years 1895 and 1896. By G. Sims Woodhead, M.D., Professor of Pathology University of Cambridge. (London: P. S. King and Son. 1901. Price 7s. 6d. net.)

This is a most elaborate and complete account of the whole matter with which it deals. In his introductory remarks Professor Woodhead states that the main portion of this Report has been in type for nearly three years, the work involved in his professorial duties having rendered him unable any earlier to find time for the revision of the proofs. The subject, however, although it has now passed somewhat beyond the stage of argument, is still one of great interest, and the Report before us will be found very useful by all who wish either to undertake similar work or to find arguments in support of scientific methods in medicine. The Report deals exclusively with the cases that were examined bacteriologically during the period between January 1st, 1895, and December 31st, 1896, but includes certain cases which were admitted before this period and some which were not discharged until after the specified period was over. During the time mentioned 27,128 cultivations were examined, and of these 24,933 could be traced and assigned to 12,172 patients. Of these cases 73·42 per cent. gave evidence of the presence of diphtheria bacilli, while in 5 per cent. of the cases it may be concluded that bacteriological examination failed to assist in the diagnosis. A table is given showing the periods over which the diphtheria bacilli persisted, from which it is seen that in one or two cases the specimens taken from the throat still contained these organisms six months after they had been first demonstrated. In two cases the bacillus was found to persist at the end of 200 days, and in 79 cases it persisted beyond 100 days.

Another matter of considerable interest is the influence of the admixture of other micro-organisms. Mixed infections were found to be always more fatal than simple diphtheria intoxications, and, contrary to what is usually stated, it was found that the presence of the staphylococcus as a complication of diphtheria appeared to be associated with a more fatal form of the disease than when a streptococcus was the secondary organism. Professor Woodhead draws attention to the marked diminution in the death rate which took place in those cases in which diphtheria occurs as a complication of scarlet fever, after they began to be treated with antitoxin, which was no doubt due to the early period at which the antitoxin was administered in these cases. Lest we should be drawn to depend too exclusively on the results of an examination by "swab," it is well to note that in 3,235 cases no diphtheria baccilli could be discovered in the swabs taken, although the patients were sent into hospital certified as suffering from diphtheria, and so far resembled that disease clinically that they were admitted as suffering from diphtheria. On the other hand, as suggesting that the non-discovery of the specific bacilli has some bearing on the probable gravity of the attack, it is to be observed that the death rate among these cases was very low. It is satisfactory to find that Professor Woodhead is able to show that the free use of antitoxin does not cause any increase in the serious complications of the disease. It does not raise the percentage of cases of albuminuria; the number of cases of adenitis appears to be distinctly reduced; and its use "has a perceptible effect in diminishing the cases of nephritis." On the other hand, there seems no doubt that in cases treated with antitoxin there is a greater percentage of joint pains than in cases not so treated. These, however, are transitory, and "are probably the result of some slight change in the blood set up by the action of the serum itself, and not by the antitoxic substance in the serum." The second part of the report is devoted to the preparation of the diphtheria antitoxin, and is by far the most full and complete account that has so far been given of this part of the business. Professor Woodhead prefaces this with a very useful survey of the present condition of our knowledge regarding the nature of the "anti-bodies" and their relation to the toxins which they have the property of neutralising, in which he shows that the antitoxin is not a modified toxin but is a product of the specially stimulated cell, a cell stimulated, that is, by the assimilation of the toxin. Another point which he insists on is that the antitoxin production is to a large extent a local affair; in other words it takes place in the tissues stimulated by the toxins which have been introduced, which go on pouring out anti-bodies for a considerable time, in fact until the blood becomes so charged with them as to check the process. The whole Report is most carefully drawn up, and is an admirable record of an immensity of good work well done.

Plague. How to Recognise, Prevent, and Treat Plague. By James Cantlie, M.A., M.B. (Aberd.), F.R.C.S., D.P.H.Lond. (London: Cassell and Co. 1900. Price 1s. 6d. net.

This is a useful pamphlet; a short monogram on plague by one who is practically acquainted with it. It was produced at the time when from the appearance of plague in Glasgow it seemed not improbable that the disease might arise in other parts of the country, and when it seemed desirable to put into the hands of the profession a short manual giving all necessary details about it, and perhaps a little hurry in its production may account for the absence of both index and table of contents, and for the loose way in which the subject is set out and divided. But the pamphlet itself is from beginning to end both readable and trustworthy. Mr. Cantlie very properly draws attention to the importance of all medical men keeping a careful watch upon the "behaviour" of all their cases whenever plague has invaded a country, for otherwise plague may stealthily creep into their midst and patients may die of it without their disease being diagnosed or even suspected. A pneumonia which carries off two or three persons in a house, typhoid or typhus fever to which a query is attachable, convulsions in children, of undetermined origin and with rapidly fatal issue, glandular fever in children, and many of other conditions and symptoms ought to suggest to the doctor's mind, if plague is in the country, that this infection may be the cause of the occurrence. In seaport towns with a large foreign trade all this is evidently necessary, but even in inland towns the danger in plague times is far from remote. The greatest danger to England at the time that Glasgow remained infected was by way of express trains by aid of which our remote inland villages might at any moment be infected by passengers coming from an infected source. It behoves everyone, then, to make himself well aware of all necessary details in regard to this disease, details which are very well put in the book before us.

